# Combining Advanced Therapies with Alternative Treatments: A New Approach to Managing Antimicrobial Resistance?

**DOI:** 10.3390/pharmaceutics17050648

**Published:** 2025-05-15

**Authors:** Greta Kaspute, Arunas Zebrauskas, Akvile Streckyte, Tatjana Ivaskiene, Urte Prentice

**Affiliations:** 1 State Research Institute Centre for Innovative Medicine, Santariskiu St. 5, LT-08410 Vilnius, Lithuania; greta.kaspute@imcentras.lt (G.K.); tatjana.ivaskiene@imcentras.lt (T.I.); 2 State Research Institute Center for Physical Sciences and Technology, Sauletekio Av. 3, LT-10257 Vilnius, Lithuania

**Keywords:** advanced therapy, propolis, essential oils, drug delivery systems, antimicrobial resistance

## Abstract

Bacterial antimicrobial resistance (AMR) represents a critical public health threat, with increasing resistance compromising the effectiveness of treatments worldwide. Resistance trends, such as fluctuating benzylpenicillin resistance in *Staphylococcus aureus*, highlight the growing urgency, with projections indicating a rise in resistance to various antibiotics, including complete resistance to gentamicin and tetracycline by 2027. Despite substantial efforts to develop new antibiotics and drug delivery systems, these approaches must undergo rigorous clinical evaluation to ensure their safety and efficacy. In parallel, alternative therapies, such as phytotherapy and apitherapy, have garnered attention for their potential in combating infections. Natural substances like tea tree essential oils and propolis, which exhibit antimicrobial properties, are being increasingly incorporated into novel drug delivery systems. However, much of the research on these materials is not new, with several studies already exploring their effectiveness. To address the escalating AMR crisis, combining advanced therapies with alternative medicine could offer a promising solution. Advanced therapy products could target bacterial genomes and enhance the effectiveness of antibiotics and natural substances. This integrated approach remains underexplored in pre-clinical and clinical trials, presenting future research opportunities to develop more effective strategies in combating AMR. Given the rapid spread of resistant infections, there is an urgent need for innovative antimicrobial agents to overcome emerging resistance mechanisms and improve diagnoses and treatments.

## 1. Introduction

Bacterial antimicrobial resistance (AMR) occurs when bacteria evolve to withstand the drugs designed to eliminate them, turning treatments into less effective ones. It has emerged as of the most pressing public health threats of the 21st century. The current situation is critical, as antibiotic resistance continues to rise. In 2019 alone, bacterial AMR was directly responsible for 1.27 million deaths worldwide and contributed to 4.95 million fatalities [1]. Between 2019 and 2023, the *Staphylococcus aureus* resistance to various antibiotics ranged from 40% to 50%. This resistance is expected to rise by an additional 18% over the next five years. While the cefoxitin resistance is expected to remain stable at 32% from 2024 to 2028, the resistance to norfloxacin (33.3% in 2023) and ciprofloxacin (16.7% in 2023) is anticipated to rise by 20%. Alarmingly, *S. aureus* could potentially significantly increase the resistance to gentamicin and tetracycline by 2027, while the amikacin resistance is projected to increase by 22.7% within the same period [2,3,4]. The emergence of AMR microorganisms has become a critical concern in both community and healthcare settings, particularly in nosocomial infections where pathogens like *Klebsiella pneumoniae*, *Acinetobacter baumannii*, and *Pseudomonas aeruginosa* exhibit multidrug resistance [5,6]. Diabetic foot ulcers and infected wound-healing sites often harbor polymicrobial biofilms [7,8], which not only delay recovery, but also foster the development of resistance to commonly used antibiotics, such as β-lactams, aminoglycosides, and fluoroquinolones [9,10]. These resistant strains are frequently isolated in clinical settings and are associated with an increased morbidity, prolonged hospital stays, and higher healthcare costs [11]. The rise in resistance is largely attributed to the overuse and misuse of antibiotics, inadequate infection control, and bacteria’s ability to acquire resistance genes through horizontal gene transfer [12].

The World Health Organization has called on countries around the world to take urgent steps to combat AMR. A primary challenge in addressing this issue is the lack of comprehensive surveillance data, particularly in regions with limited monitoring systems [1]. For example, China has implemented its National Action Plan (2022–2025). This plan adopts a One Health approach, integrating human health, veterinary, agriculture, and environmental sectors to tackle the crisis. The plan focuses on reducing unnecessary antibiotic use, strengthening surveillance networks, advancing research and development for novel treatments, establishing national standards for antimicrobial susceptibility testing, and enhancing international collaboration [13].

**Figure 1:**
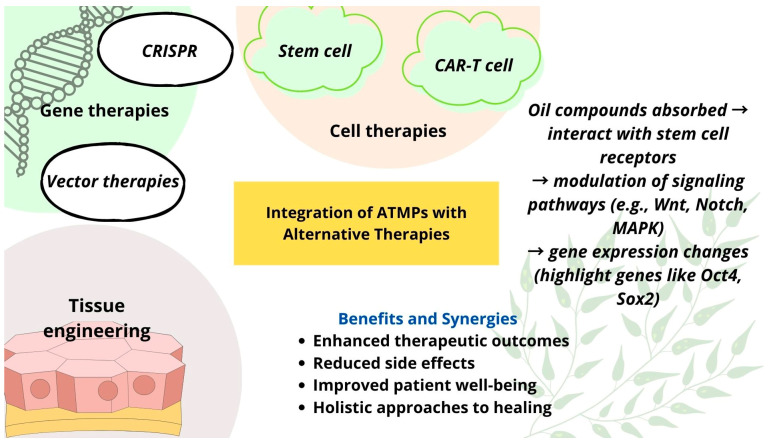
A graphical summary of the integration of ATMPs with alternative therapies.

Given the urgent need for novel solutions, this article explores the potential of combining advanced therapies with alternative medicine to manage AMR. Figure 1 shows a graphical summary illustrating the integration of advanced therapy medicinal products (ATMPs) with alternative therapeutic approaches. ATMPs, including gene therapy, somatic cell therapy, and tissue-engineered products, represent cutting-edge innovations with a promising potential for treating conditions with limited therapeutic options [14]. These therapies could play a key role in combating AMR. Gene-editing technologies, such as CRISPR [15], can be used to modify bacteria or viruses, making them more susceptible to existing antibiotics or removing resistance genes from microbial populations. Additionally, cell-based approaches could be developed to engineer cells capable of detecting and eliminating resistant bacteria in the body [16].

Alongside ATMPs, alternative medicine approaches may contribute to the fight against AMR [17]. Natural substances with strong antibacterial properties, such as propolis [18,19,20] and essential oils [21,22], and their combination with nanotechnology-based delivery systems—such as zinc oxide nanoparticles (ZnO NPs) [23]—hold promise as complementary strategies. Integrating advanced therapies with alternative medicine may uncover innovative ways to address AMR and mitigate its growing impact on global health. Despite their potential, the implementation of ATMPs and alternative therapies in AMR management faces significant challenges, including high development costs, complex regulatory pathways, and difficulties in targeted delivery. Additionally, many alternative treatments lack robust clinical validation, limiting their immediate applicability in clinical settings.

## 2. Trends in Antimicrobial Drug Formulations

The rapid spread of resistant microbial infections underscores the urgent need for new antimicrobial agents, as resistance hinders effective diagnoses and treatments. Antibiotics function by inhibiting cell wall or protein synthesis, disrupting bacterial membranes, and causing intracellular component loss [24]. The inappropriate exposure to sublethal drug doses has led to mutations that contribute to the development of antimicrobial resistance in diseases like tuberculosis [25]. Furthermore, synthetic small molecule libraries have struggled to produce effective antibiotics, as these drugs often require complex, larger structures with multiple stereocenters, unlike the simpler compounds typically found in synthetic libraries [26]. Novel drug formulation strategies are continuously being developed to address these challenges. For example, scientists have applied click chemistry methods to examine differences in chemical structures and explore new opportunities to control infections. Di-branched and tetra-branched analogues of the lipopeptide battacin were synthesized using thiol-maleimide and 1,2,3-triazole click chemistry, showing enhanced antimicrobial activity against drug-resistant bacteria and fungi in the low micromolar range. The di-branched peptides, particularly Peptide 12, demonstrated improved membrane lysis, faster killing kinetics, antibiofilm activity, and strong proteolytic stability compared to the monomer peptides, with up to 40-fold higher selectivity for bacteria and fungi. These findings suggest that dual branching through triazole click chemistry is an effective strategy. This method could be used to develop potent and stable antimicrobial dendrimeric peptides as new peptide antibiotics [27]. Zwitterionic polymers like poly(sulfur ylides) have shown great potential in preventing harmful bacterial biofilms, offering a safer alternative to commonly used materials like polyethylene glycol. These compounds can disrupt bacterial membranes without harming human cells, showing that they are both effective and selective. This makes ylide-based materials promising for creating new protective coatings used in medicine, drug delivery, and nanotechnology [28].

Bacterial infections, especially in low-income regions, remain a major global health threat, exacerbated by antibiotic overuse leading to multidrug-resistant strains. This has spurred the development of nanomaterials with intrinsic antibacterial properties or targeted drug delivery capabilities, offering promising passive and active strategies for more effective and selective antibacterial treatments [29]. Nanotechnology-based approaches are being integrated to enhance drug stability, targeted delivery, bioavailability, and controlled drug release while achieving antimicrobial effects [30,31,32,33]. Combining multiple antimicrobial agents or incorporating alternative substances can help overcome resistance mechanisms [17], while biofilm-targeting formulations play a crucial role in combating AMR. The metal–organic framework NG-MOF-5@LNs demonstrated strong antimicrobial activity, confirmed by MTT assay and inhibition zone studies [34]. Similarly, carboxymethylcellulose-based hydrogels delivering *Justicia adhatoda* showed an enhanced mechanical strength and antimicrobial and antioxidant properties, and they effectively reduced biofilm formation [35]. Table 1 shows some trends in antimicrobial drug delivery formulations, highlighting the emerging technologies and their associated therapeutic advantages. Moreover, scientists have evaluated a triple antibiotic combination—meropenem (MEM), a novel MBL inhibitor (InC58), and an SBL inhibitor (avibactam)—for its broad-spectrum activity against carbapenemase-producing bacteria. The combination was more effective than dual therapies, showing activity against most MBL- and SBL-producing strains, except for some resistant *P. aeruginosa* and *Acinetobacter baumannii*. Resistance emerged through mutations affecting porin and copper transport, with associated fitness costs and cross resistance, highlighting both the promise and challenges of this new therapeutic strategy [36].

**Figure 2:**
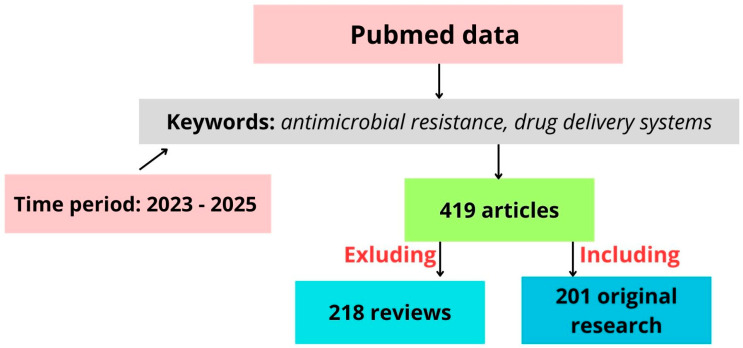
Schematic representation of literature selection process used in this study.

According to data from PubMed for the 2023–2025 period, 419 articles were identified on antimicrobial drug delivery and novel systems (Figure 2). Nearly half (218) of these were review articles, indicating that the topic has been well analyzed and is an active area of new research. The reviews summarize the ideas for controlling AMR, which poses a critical threat to human, animal, and environmental health. The authors suggest the need for integrated, data-driven systems like AMR-X to transform routine clinical data into actionable insights for optimal antimicrobial use [44]. One promising strategy to combat AMR is the use of lipid-based nanocarriers (liposomes), which effectively target biofilm-forming, multidrug-resistant, Gram-negative and Gram-positive bacteria [45]. Recent advancements in nano-drug delivery systems and antimicrobial materials show potential in reducing resistance, enhancing targeted drug delivery, and extending antibiotic efficacy. Challenges remain in optimizing their design and application across diverse bacterial strains [44,45,46,47]. As one more example, antimicrobial peptides are naturally occurring polypeptides found in both prokaryotes and eukaryotes that play a key role in immunity by targeting a wide range of pathogens, offering potential as alternatives to antibiotics amid rising resistance. However, their clinical use is limited by issues such as cytotoxicity and instability in the blood, though recent advances aim to overcome these challenges compared to other alternatives like bacteriophages, traditional medicine, and probiotics [48]. Therefore, the true novelty in the field of AMR lies in the potential to combine advanced therapy practices—such as gene editing and cell-based therapies—with alternative treatments. This approach, which integrates bacterial modifications with natural substances as complementary or combined treatments, remains largely unexplored.

## 3. Advanced Therapy

ATMPs can be categorized into three main types: gene therapy, somatic cell therapy, and tissue-engineered medicines [49,50]. With advancements in gene vectors, the emergence of chimeric antigen receptor T-cell immunotherapy, and breakthroughs in genome editing technologies, gene therapy has once again become a central focus in disease treatment [51]. Gene therapy medicines contain recombinant genes inserted into the body to treat diseases such as genetic disorders, cancer, or chronic diseases [49]. Somatic cell therapy (SCC) medicines involve cells or tissues manipulated to alter their biological functions, and are used to help treat, diagnose, or prevent diseases [52]. Tissue-engineered medicines contain modified cells or tissues to repair, regenerate, or replace damaged human tissue [53]. Some ATMPs also combine medical devices, known as combined ATMPs, including cells within a biodegradable scaffold [49].

The development of innovative gene editing and targeting strategies to fight bacterial infections has been a key focus for researchers since the discovery of antibiotics. Initially found in archaea and bacteria, CRISPR/Cas was identified as a mechanism for adaptive immunity. Over the past decade, CRISPR/Cas has been adapted for gene editing and genetic engineering. CRISPR/Cas gene editing and base editing are two essential techniques for precisely modifying an organism’s DNA [54,55]. The CRISPR/Cas bacterial defense mechanism operates in three distinct stages:

I. Adaptation stage: During this phase, spacer sequences are acquired from invading genetic material, such as viruses or plasmids.
II. Expression stage: In this stage, the CRISPR array is transcribed to generate crRNA (CRISPR RNA), which, along with the Cas protein, forms the essential components for targeting invading DNA.
III. Interference stage: The mature crRNA binds to the Cas protein, forming a nucleic acid–protein complex. This complex can then recognize and bind to complementary sequences in the target nucleic acid, triggering endonuclease activity that cleaves and degrades the foreign genetic material.

**Figure 3:**
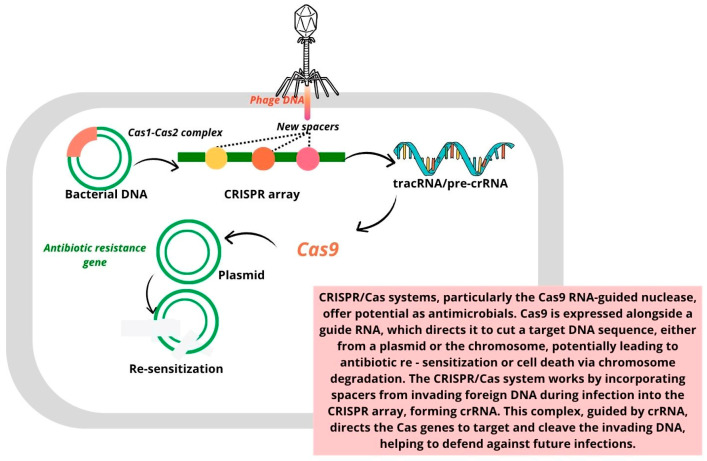
Eliminating antibiotic-resistant genes in bacteria using CRISPR/Cas systems to re-sensitize bacteria to antibacterial agents [54,56,57,58].

This targeted cleavage capability allows the CRISPR system to be engineered to eliminate specific drug resistance genes, thus controlling horizontal gene transfer and helping limit the spread of antibiotic resistance [54]. Figure 3 shows the use of CRISPR/Cas systems to eliminate antibiotic-resistant genes in bacteria, thereby re-sensitizing them to antibacterial agents [54,56,57,58].

Another group of ATMPs are stem cells. It is important to mention that not all stem cells can be categorized as ATMPs [49]. Scientists have evaluated the relationship between the pre-treatment SCC and the success of antibiotic treatment for intramammary infections in dairy cattle. The results showed that a lower pre-treatment SCC was associated with higher cure rates, with cows that failed to cure having a significantly higher SCC both before and after treatment. These findings suggest that developing SCC-dependent treatment protocols could improve the cure rates and promote better mammary health in dairy cattle [59]. One more study identified mesenchymal stem cell (MSC) antimicrobial properties, including the ability to directly kill bacteria and secrete factors that disrupt and prevent biofilm formation. These secreted factors enhance the effects of antibiotics and stimulate host immune responses, such as increased phagocytosis and neutrophil activity [60]. Human bone-marrow-derived MSCs exhibit direct antimicrobial activity partly through the secretion of the cathelicidin peptide hCAP-18/LL-37, whose expression increases after bacterial exposure [61]. In a mouse model of *E. coli* pneumonia, MSC treatment reduced the bacterial load in the lungs and bronchoalveolar lavage (BAL) fluid, while blocking LL-37 impaired this effect. Additionally, BAL fluid from MSC-treated mice showed greater antimicrobial activity than that from control mice, confirming LL-37’s key role in bacterial clearance [61]. In a *nu/nu* mouse model of a chronic *S. aureus* infection, activated MSCs combined with antibiotics significantly reduced the bacterial load and improved wound healing [60]. Examples of animal studies provide valuable insights into biological mechanisms and potential therapeutic targets, but their findings do not always translate directly to humans due to species-specific differences. Therefore, the results from animal research should be interpreted with caution and validated in well-designed human studies before clinical application.

Tissue engineering aims to create biomimetic 3D cellular microenvironments using a combination of natural and synthetic biomaterials to guide tissue regeneration, with scaffolds tailored to mimic the in vivo cellular environment. Due to the growing problem of antibiotic resistance, alternative antimicrobial agents, including metal ions, antimicrobial peptides, and biometals, are being explored for their potential to enhance tissue regeneration while preventing infections in scaffold implantations [62]. Polycaprolactone-based biomaterials for bone tissue engineering can be enhanced with antimicrobial agents to prevent bacterial and fungal adhesion, as well as biofilm formation, on the scaffolds. These scaffolds, which can be combined with calcium phosphates, offer favorable properties like biocompatibility, biodegradability, and ease of shaping, promoting cell attachment and osteogenesis. Antimicrobial agents such as silver and essential oils, when used at lower concentrations, have been shown to effectively counteract microbial growth and biofilm formation, with further in vivo studies needed to validate their safety and efficacy for bone healing [63].

The application of ATMP therapies for the treatment of microbial infections includes both direct cell therapy and the use of cellular products (e.g., extracellular vesicles (EVs) and exosomes). Cell therapy involves, for example, the use of stem cells in cases of bacterial infection. Various studies have shown that the application of MSCs in different models demonstrates both a direct bactericidal effect through the secretion of various bactericidal substances and an indirect immunomodulatory effect, which involves the activation of various immune mechanisms [60,64,65,66,67,68]. Thus, MSCs, in studies where they were used alone as well as in combination with antibiotics, showed a statistically significant antibacterial effect. Extracellular vesicles secreted by stem cells also demonstrate antibacterial efficacy. Research indicates that the immunomodulatory effect of substances (mRNA, proteins) carried by EVs can be an effective factor in combating bacteria [69]. EVs, particularly those derived from MSCs, play a critical role in intercellular communication and have shown therapeutic potential in treating various infectious diseases by delivering antimicrobial agents, enhancing immune responses, and promoting tissue repair. MSC-derived exosomes (MSC-Exos) also show promise in wound healing and reducing pathological scarring due to their immunomodulatory effects on different immune cells involved in inflammation and tissue regeneration. These findings highlight the potential of MSC-EVs and MSC-Exos as innovative treatments for both infections and scar-related complications [66,68].

## 4. Drug Delivery Systems

Targeted and responsive drug delivery systems can be designed using internal stimuli (pH, temperature, enzymes, redox) or external stimuli (light, magnetic fields, ultrasound). Since infection sites typically have a lower pH due to anaerobic glycolysis and negatively charged bacterial cell walls, these characteristics can be leveraged for precise antibiotic delivery [70]. Bacteria enhance their resistance by forming biofilms that act as physical barriers. Developing drug delivery systems that respond to the biofilm microenvironment offers a promising strategy to overcome this challenge [71,72]. Natural product screening has been the most successful approach for discovering new antibiotics, but finding novel compound structures has become increasingly difficult due to the frequent rediscovery of existing antibiotics [26].

Overcoming AMR requires innovative approaches beyond conventional drug delivery. Multifunctional delivery systems have been designed to integrate various substances, enabling their controlled release, antibacterial activity, and additional biomedical applications such as wastewater treatment. An example of such a system is multifunctional gallic acid cross-linked zein composite fibers integrating poly(N-(4-aminophenyl)methacrylamide)-carbon nano-onions for phosphate removal. The fibers exhibited an exceptional phosphate adsorption capacity (2500 mg/g at a pH of 7.0), a high stability across 13 adsorption cycles, and pH-responsive azithromycin release over 18 days. Additionally, they demonstrated strong antibacterial properties and an enhanced mechanical strength [73]. Combining gene therapy with natural antimicrobial agents such as essential oils or propolis could offer a powerful dual-action strategy. Gene therapy can target bacteria at the genetic level by disrupting resistance mechanisms, silencing virulence genes, or enhancing host immune responses. Following this genetic disruption, natural substances can deliver the final blow by exerting their inherent antibacterial properties.

**Figure 4:**
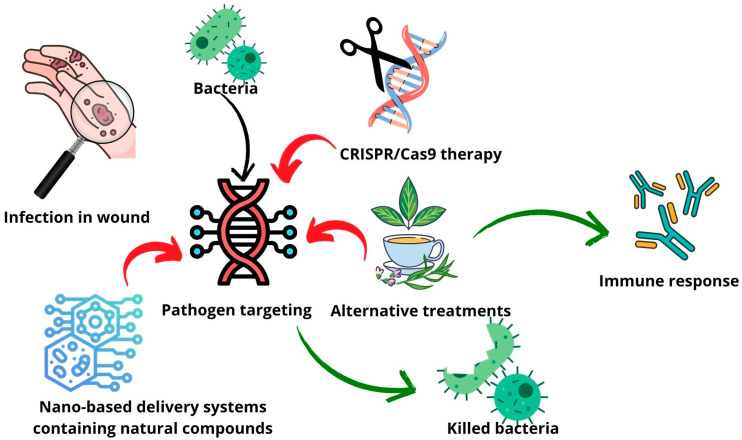
Schematic representation of possible alternative treatment and advanced therapy combination strategy.

Incorporating these natural compounds into advanced drug delivery systems, such as liposomes, exosomes, or NPs, enhances their bioavailability, stability, and targeted delivery (Table 2). Numerous studies have highlighted the biological effects of essential oils and propolis in fighting infections. Propolis, which is rich in bioactive compounds like flavonoids and phenolic acids, has been extensively researched for its antimicrobial properties [74,75,76,77,78] and is already integrated into various delivery platforms [79,80,81,82,83,84]. Essential oils, which are gaining increasing scientific attention, demonstrate broad-spectrum antibacterial activity by disrupting bacterial membranes, inhibiting quorum sensing, and interfering with metabolic pathways and incorporation in drug delivery systems [85,86,87,88,89,90,91,92,93,94]. For example, cinnamaldehyde-enhanced gelatin (cinnamyl-gelatin), with improved antibacterial and antioxidant properties, targeted multi-drug-resistant bacteria from chronic wounds. The modified gelatin showed an enhanced thermal stability, a reduced solubility, and strong antimicrobial and radical scavenging activities. As a result, it promoted fibroblast proliferation, suggesting its potential for use in antibiotic-free wound dressings and tissue engineering applications [95]. Propolis extracts from Bihor County, Romania, were analyzed to assess the relationship between their polyphenolic profiles and biological activities, using varying concentrations of ethanol and water as solvents. UHPLC-MS identified 21 polyphenolic compounds, with 50% ethanolic extracts yielding the highest content and the strongest antioxidant activity, as shown by DPPH and ABTS assays. All the extracts, including the aqueous ones, exhibited antibacterial and antifungal effects, with a chemometric analysis confirming key polyphenols as the major contributors to the antioxidant capacity [96]. Propolis harvested from Germany, Ireland, and the Czech Republic revealed a rich phytochemical diversity and notable antioxidant and antibacterial properties, particularly in the Czech and Irish samples. Ethanol extracts of propolis showed moderate antimicrobial effects and demonstrated synergistic interactions with antibiotics like vancomycin and oxacillin against resistant pathogens such as *Bacillus subtilis* and *Streptococcus pyogenes* [19]. By harnessing the synergy between gene therapy and bioactive natural substances, this strategy holds great promise in tackling AMR while minimizing side effects and the environmental impact (Figure 4).

Incorporating metal–organic NPs into drug delivery systems can enhance their antimicrobial efficacy. NPs, particularly metallic types such as silver (AgNPs), ZnO, and copper oxide (CuO), have demonstrated potent antimicrobial properties by generating reactive oxygen species, disrupting microbial membranes, and interfering with DNA replication [97]. Polymeric NPs offer targeted drug delivery capabilities, enhancing the bioavailability and stability of antimicrobial agents while minimizing the toxicity to host tissues [98]. Recent studies have also explored the synergistic effects of combining nanoparticles with enzymes like lysozymes, which can degrade biofilms and disrupt microbial cell walls, further enhancing the antimicrobial efficacy [99]. These novel approaches are gaining traction as alternatives to conventional antibiotics, especially in treating chronic wounds, hospital-acquired infections, and resistant biofilms [100]. Table 2 shows some examples of drug delivery systems and their activities and applications. ZnO NPs, known for their strong antibacterial properties, are an excellent choice for combination with natural substances to develop advanced antimicrobial drug delivery platforms. ZnO NPs exert their antimicrobial effects by generating reactive oxygen species, disrupting bacterial membranes, and interfering with essential cellular processes [101,102]. For example, a metal–organic-framework-based drug delivery platform, MV@ZIF-8, was used to modify titanium implants. It improved their antibacterial and osteogenesis-promoting properties. The sustained release of Zn^2^⁺ and methyl vanillate (MV) caused oxidative damage to *E. coli* and *S. aureus*, inhibiting bacterial proliferation while upregulating oxidative stress and DNA damage response genes. Additionally, the MV@ZIF-8 coating promoted the osteogenic differentiation of human bone mesenchymal stem cells by activating the Wnt/β-catenin signaling pathway, offering a promising solution for bone tissue engineering [103]. Another study aimed to enhance the efficiency of molnupiravir (MOL) for COVID-19 therapy. It was loaded onto self-assembled biomolecule NPs (CNC@Pyc) decorated with ZnO NPs. The modified drug formulations demonstrated an improved release efficiency, with CNC@Pyc.MOL.ZnO achieving 76–78% drug release over 19 h, along with enhanced antioxidant and anti-inflammatory properties. An antiviral evaluation against the 229E virus showed promising inhibition rates (37.6% at 800 µg/mL) [104].

When combined with bioactive natural compounds like essential oils or propolis, ZnO NPs can create a synergistic effect, improving both the stability and targeted delivery [104,105,106,107]. The combination of ZnO NPs with essential oils (e.g., citronella, orange, thyme, lavender, grapefruit, bergamot, cinnamon, rosemary, minzol, or limette) demonstrated a synergistic antimicrobial effect, with volatile compounds effectively retained on the NP surface for prolonged activity. Antibacterial assays confirmed that the efficacy depended on the concentration of the loaded oil, highlighting the role of the formulation [108]. Integrating these components into nanocarriers such as liposomes, exosomes, or polymeric NPs can further enhance their therapeutic potential, offering a promising strategy to combat AMR and bacterial infections more effectively. Using an in situ sol–gel technique, propolis-loaded ZnO nanoarchitectonics (PP/ZnO NPs) were synthesized. This system improved the performance of a collagen–chitosan gel in wound healing. Characterization revealed a shift from agglomerated ZnO NPs to needle-like and sheet-like morphologies with increased surface negativity upon propolis incorporation. The nanocomposite gel, loaded with 10% PP1/ZnO NPs, exhibited strong antimicrobial activity against *E. coli* and significantly improved wound healing through enhanced collagen deposition, faster closure rates, and re-epithelialization [107]. Soluble soybean polysaccharide (SSPS)-based composite films incorporating nano-ZnO and tea tree essential oil (TTO) were developed, demonstrating improved physical, mechanical, and functional properties. The SSPS/TTEO/ZnO film exhibited an enhanced water resistance, thermal stability, UV-blocking ability, and antioxidant activity, while also showing significant antibacterial effects against *E. coli* and *S. aureus* [109]. More complex delivery systems containing phyto-materials and ZnO NPs have also been developed. For example, scientists constructed a hybrid nanoplatform delivery system integrating chemo- and photodynamic therapy using ZnO NPs, porphyrin (POR), alginate (ALG), and berberine (BER) to combat microbial infections. The optimized ZnO@ALG-POR/BER nanoformulation exhibited a asuperior loading capacity (22.2 wt%) and entrapment efficiency (95.2%), leading to 100% bacterial and fungal growth suppression, especially against *E. coli* and *C. albicans*, when combined with laser irradiation. Cytotoxicity assessments showed a moderate biocompatibility, with ZnOBER@ALG-POR being the most biocompatible, causing only ~9% inhibition of RPE1 cells [110].

**Table 2:** Examples of drug delivery systems and their activities and applications.

Drug Delivery System	Activity	Application
OnG6 MOFs serve as mesoporous metal–organic frameworks capable of loading multiple antibiotics, including isoniazid and ciprofloxacin [111].	MOFs loaded with ciprofloxacin exhibited strong antimicrobial activity against *S. aureus* and *E. coli*.	These MOFs are designed for treating bacterial infections, particularly tuberculosis, by releasing active drugs like ASA in vivo and enhancing the delivery efficiency.
A smart, bacteria-responsive carrier system (CMC-EFT@ZIF-8) was developed using ZIF-8 and carboxymethyl cellulose to deliver the antibiotic ceftiofur [112].	The system demonstrated enhanced drug release under acidic and cellulase-rich conditions, resulting in the 99% elimination of *P. aeruginosa* in vitro.	This delivery platform showed strong therapeutic potential in a mouse skin wound model, offering a promising approach for treating resistant bacterial infections.
A thyme-oil-based nanoemulsion (NE) was developed to enhance the stability and bioavailability and control the release of the volatile and easily oxidized essential oil compound thymol [113].	The NE showed potent antibacterial effects against *B. subtilis*, *E. coli*, *P. aeruginosa*, and *S. aureus*, and antitumor activity by inducing apoptosis and cell cycle arrest in HepG2 liver cancer cells.	This nanoemulsion system offers a dual-function therapeutic platform for bacterial infection control and cancer treatment, making it a promising alternative to conventional therapies.
Lavender oil was incorporated into niosomes to enhance delivery, reduce volatility, and improve cellular compatibility in biomedical applications [114].	The niosomes maintained a high cell viability across the tested concentrations in adipose-derived stem cells and myometrial cells, indicating a low cytotoxicity and biocompatibility.	This system showed strong potential for regenerative medicine and pharmaceutical therapies, offering a safe and natural alternative for future biomedical formulations.
Solid lipid microparticles (SLM) were developed using hardfat and palm oil carriers via spray-chilling to encapsulate cinnamon bark oleoresin (CO), protecting its bioactive components from degradation [115].	The SLMs exhibited strong antimicrobial activity, maintaining effective inhibition against *Candida pseudointermedia* and *Penicillium paneum*, with an enhanced performance over a 28-day period.	These lipid-based microparticles offer a stable and controlled-release system for natural antimicrobials, making them suitable for applications in food preservation, pharmaceuticals, and possibly topical therapeutics.
A bioabsorbable, controlled-release nanoemulgel of quercetin was developed using cinnamon oil, tween 80, Carbitol^®^, and poloxamer 407 as the base, with the aim of enhancing its solubility and bioavailability for periodontitis treatment [116].	The nanoemulgel showed a significant drug release of 92.4% quercetin within 6 h, far surpassing the release from a pure quercetin-loaded gel (<3% release), indicating efficient drug delivery.	This nanoemulgel system holds potential as an effective therapeutic delivery platform for treating periodontitis, improving the clinical outcomes by enhancing quercetin’s antimicrobial and anti-inflammatory effects.
A Pickering emulsion was developed using zein–tannic acid complexes to co-load tannic acid and cinnamon essential oil, enhancing the interfacial stability and controlled release [117].	The optimized formulation exhibited strong antimicrobial activity against the spoilage organisms *Pseudomonad paralactis* MN10 and *Lactobacillus sakei* VMR17.	This system presents a novel approach for food preservation, enabling the effective delivery of multiple natural antimicrobials through a stable, bio-based emulsion interface.
Propolis-based chitosan varnishes were formulated in 5%, 10%, and 15% concentrations to ensure controlled release and strong adhesion to tooth surfaces [118].	The system demonstrated strong antimicrobial activity—comparable or superior to chlorhexidine—against cariogenic biofilm-forming bacteria, with the sustained release of active compounds for over one week.	These formulations show promise for preventive dental care, specifically in managing and preventing dental caries, and warrant further clinical investigation.
A targeted NP carrier system (PBCA-NP) functionalized with polysorbate 80 to adsorb apolipoprotein E was developed to deliver propolis across the blood–brain barrier [119].	The propolis-loaded PBCA-NPs exhibited significant antifungal activity against *Cryptococcus neoformans* in vitro and reduced fungal virulence and burden in both *Galleria mellonella* and mouse models.	This system demonstrates strong potential for treating cerebral cryptococcosis, overcoming bioavailability issues and targeting the central nervous system through a blood–brain-barrier-crossing mechanism.
TTO was formulated into dry powder inhalers using β-cyclodextrin inclusion complexes (TTO-β-CD) to enable effective pulmonary delivery [120].	TTO-β-CD powders showed superior antipneumonic activity compared to TTO alone, and performed comparably to fluconazole and penicillin in rat models of fungal and bacterial pneumonia, respectively, through antimicrobial and anti-inflammatory mechanisms.	The TTO-β-CD dry powder inhalers offer a promising inhalable therapy for managing fungal and bacterial pneumonia, with their advantages including s high lung deposition, stability, and self-administration suitability.
Hydrogels incorporating TTO-loaded nanocapsules and nanoemulsions were formulated using Carbopol Ultrez, offering stable and skin-compatible topical delivery [121].	These formulations showed significant anti-inflammatory (antiedematogenic) effects following UVB exposure, and enhanced wound healing, with nanocapsule-based hydrogels outperforming other treatments in reducing the wound area.	This study supports the topical use of nanostructured tea tree oil hydrogels for managing skin inflammation and cutaneous wound repair, demonstrating their potential in dermatological therapy.
Air-filled lysozyme microbubbles functionalized with gold NPs and alkaline phosphatase [122].	Enhanced antimicrobial activity against *M. lysodeikticus* and effective biosensing of paraoxon in aqueous solutions.	The use of air-filled lysozyme microbubbles functionalized with gold NPs and alkaline phosphatase includes antimicrobial therapy and biosensing, specifically for detecting paraoxon in aqueous solutions.
Injectable paste composed of mannitol, chitosan, and polyethylene glycol designed for localized antibiotic delivery [123].	Mannitol reactivated dormant *S. aureus* persister cells, enhancing the antibiotic susceptibility, and when combined with vancomycin or amikacin in a paste formulation, it extended drug release by up to 7 days and reduced biofilm viability by up to 95.5%, with mannitol alone also contributing to biofilm disruption.	This represents a promising adjunctive treatment for musculoskeletal infections, especially where biofilm-forming bacteria like *S. aureus* are involved.

## 5. Conclusions

Antimicrobial resistance (AMR) has been a longstanding issue for centuries. Even with the discovery of the first antibiotics, the potential for AMR was a concern that should have been considered from the outset. Much research has focused on discovering new antibiotics or innovative delivery systems for existing ones. While many of these approaches show promise in vitro and in vivo, their efficacy and safety must be thoroughly evaluated in clinical trials before they can be widely adopted.

In addition to synthetic antibiotics, alternative medicine treatments, such as phytotherapy and apitherapy, have gained attention for their potential in combating infections. Substances like essential oils and propolis, which possess antimicrobial properties, have become more widely recognized. As the interest in natural materials grows, an increasing focus is on incorporating them into novel drug delivery systems. However, much of the research into natural materials is not entirely new, as many studies have already explored their antimicrobial effects in recent years.

To make further advancements, combining seemingly distinct therapeutic approaches—such as advanced therapy and alternative medicine—could offer a new direction. Advanced therapy products hold potential for targeting bacterial genomes and for strengthening the effects of antibiotics and natural materials. This combination of therapies remains largely unexplored and warrants further research. By integrating modern and natural treatment strategies, there is a way to open new pathways for addressing the pressing issue of AMR. Future perspectives should prioritize translational research and the clinical validation of these combination therapies to ensure their safety, scalability, and long-term effectiveness against AMR.

## Figures and Tables

**Table 1:** Innovative trends in antimicrobial drug delivery formulations (2023–2025). The keywords used for the search were the following: antimicrobial resistance; drug delivery systems.

Example	Details	Reference
**Biosurfactant-based nanoemulsions**	Broad-spectrum antibacterial activity and significant antibiofilm effects against *E. coli* and *S. aureus.*	[37]
**Silver-poly(ionic liquid) nanocomposite**	Antibacterial activity against *E. coli* and *S. aureus*.	[38]
**Vancomycin hydrochloride-loaded multivesicular liposomes**	An encapsulation efficiency of over 90%, with the drug released over a sustained period—up to 19 days—compared to 6–8 h for free vancomycin hydrochloride. Effective antibacterial activity against osteomyelitis-causing pathogens.	[39]
**Hybrid nanocomposite (Cs@Pyc.SOF) of sofosbuvir, pycnogenol, and chitosan NPs**	An 83% drug-loading efficiency and a controlled release of up to 94% over 48 h.	[40]
**A self-nanomicellizing solid dispersion system using Soluplus^®^**	Encapsulated narasin achieved a 100-fold increase in solubility, demonstrated superior skin penetration compared to free narasin, and exhibited strong antibacterial activity.	[41]
**Naringin-loaded Zn–organic framework 5 (NG-MOF-5) coated with liponiosomes (LNs)**	The NG-MOF-5@LNs exhibited monodispersed spherical particles with excellent antimicrobial activity, an IC50 of 21 µg/mL against MCF-7 breast cancer cells, and a significant apoptosis effect of 68.2%, as indicated by the MTT assay and inhibition zone results.	[34]
**Cationic and anionic PLGA–cholesterol hybrid NPs for the intracellular delivery of benznidazole**	Demonstrated enhanced trypanocidal activity against intracellular amastigotes and a superior performance in anionic NPs, attributed to effective internalization and endo-/lysosomal residence.	[42]
**Carboxymethylcellulose-based hydrogels delivering *Justicia adhatoda***	Improved mechanical properties, antimicrobial activity, reduced biofilm formation, and antioxidant effects.	[35]
**Halloysite nanotubes combined with the iron-chelating capabilities of kojic acid**	Strong antibacterial activity against all tested pathogens and successfully loaded resveratrol and curcumin as potential drug carriers, offering dual functionality as both an antimicrobial agent and a drug delivery system.	[43]

## Abbreviations

The following abbreviations are used in this manuscript:

AMR	antimicrobial resistance
ATMPs	advanced therapy medicinal products
EV	extracellular vesicle
MOL	molnupiravir
MSC	mesenchymal stem cells
MV	methyl vanillate
NPs	nanoparticles
SCC	somatic cell therapy
SLM	solid lipid microparticles
TTO	tea tree oil
ZnO	zinc oxide
